# Predictive and prognostic nomogram models for liver metastasis in colorectal neuroendocrine neoplasms: a large population study

**DOI:** 10.3389/fendo.2024.1488733

**Published:** 2025-01-07

**Authors:** Xiao Lei, Yanwei Su, Rui Lei, Dongyang Zhang, Zimeng Liu, Xiangke Li, Minjie Yang, Jiaxin Pei, Yanyan Chi, Lijie Song

**Affiliations:** ^1^ Department of Oncology, The First Affiliated Hospital of Zhengzhou University, Zhengzhou, China; ^2^ Henan Neuroendocrine Tumor Medical Center, The First Affiliated Hospital of Zhengzhou University, Zhengzhou, China; ^3^ Department of Endocrinology, Zhoukou First People‘s Hospital, Zhoukou, China; ^4^ School of Basic Medical Sciences, Xinxiang Medical University, Xinxiang, China

**Keywords:** colorectal neuroendocrine neoplasms, liver metastases, overall survival, nomogram, SEER, prognostic factors, risk factors

## Abstract

**Background:**

In recent years, the incidence of patients with colorectal neuroendocrine neoplasms (CRNENs) has been continuously increasing. When diagnosed, most patients have distant metastases. Liver metastasis (LM) is the most common type of distant metastasis, and the prognosis is poor once it occurs. However, there is still a lack of large studies on the risk and prognosis of LM in CRNENs. This study aims to identify factors related to LM and prognosis and to develop a predictive model accordingly.

**Methods:**

In this study, the Surveillance, Epidemiology, and End Results (SEER) database was used to collect clinical data from patients with CRNENs. The logistic regression analyses were conducted to identify factors associated with LM in patients with CRNENs. The patients with LM formed the prognostic cohort, and Cox regression analyses were performed to evaluate prognostic factors in patients with liver metastasis of colorectal neuroendocrine neoplasms (LM-CRNENs). Predictive and prognostic nomogram models were constructed based on the multivariate logistic and Cox analysis results. Finally, the capabilities of the nomogram models were verified through model assessment metrics, including the receiver operating characteristic (ROC) curves, calibration curve, and decision curve analysis (DCA) curve.

**Results:**

This study ultimately encompassed a total of 10,260 patients with CRNENs. Among these patients, 501 cases developed LM. The result of multivariate logistic regression analyses indicated that histologic type, tumor grade, T stage, N stage, lung metastasis, bone metastasis, surgery, and tumor size were independent predictive factors for LM in patients with CRNENs (*p* < 0.05). Multivariate Cox regression analyses indicated that age, primary tumor site, histologic type, tumor grade, N stage, tumor size, chemotherapy, and surgery were independent prognostic factors (*p* < 0.05) for patients with LM-CRNENs. The predictive and prognostic nomogram models were established based on the independent factors of logistic and Cox analyses. The nomogram models can provide higher accuracy and efficacy in predicting the probability of LM in patients with CRNENs and the prognosis of patients with LM.

**Conclusion:**

The factors associated with the occurrence of LM in CRNENs were identified. On the other hand, the relevant prognostic factors for patients with LM-CRNENs were also demonstrated. The nomogram models, based on independent factors, demonstrate greater efficiency and accuracy, promising to provide clinical interventions and decision-making support for patients.

## 1 Introduction

Neuroendocrine neoplasms (NENs) are infrequent and highly heterogeneous tumors typically originating from polypeptide neurons and neuroendocrine cells. NENs occur in almost any body organ, but the digestive system is the most common site of occurrence, especially the pancreas and gastrointestinal tract (1). According to the Ki-67 proliferation index and mitotic count, neuroendocrine tumors are classified into well-differentiated neuroendocrine tumors (NET G1, G2, G3), poorly differentiated neuroendocrine carcinomas (NEC), and mixed neuroendocrine-non-neuroendocrine tumors (2). In recent years, some epidemiological studies have shown that the global incidence of gastroenteropancreatic neuroendocrine neoplasms (GEP-NENs) has continuously increased (3–6). Particularly, the incidence of CRNENs increases significantly (5, 7). The development and widespread use of endoscopic techniques has led to more patients being diagnosed, which may be the primary reason for this trend (8). NENs demonstrate significant heterogeneity (9), different from common tumors for cellular origin (10), biological behavior (11), pathological features (12), clinical manifestations (13), and therapeutic modalities (13).

Although most NENs are indolent tumors, some advanced NENs and NEC are more malignant and invasive (14). Some studies demonstrated that over 50% of patients already have distant metastases when diagnosed, and the liver is the most common organ of distant metastasis (15, 16). Once LM occurs, the survival rate of patients will significantly decrease (17). Consequently, clinicians accord considerable significance to LM. Currently, the treatment alternatives for LM-CRNENs mainly include local therapies, systemic therapies, surgical interventions, and liver transplantation et al (18), but the efficacy of these treatments has yet to be definitively determined. Thus, it is crucial to identify the risk factors and prognostic indicators related to LM and to undertake timely interventions. Some studies indicated that among NENs located in different sites, the probability of metastasis in the rectum and colon is second only to that in the pancreas and small intestine (18). However, now, most studies focused on distant metastases of GEP-NENs or metastasis to other sites, such as the lungs, lymph nodes, etc. (19–21), with little research specifically studying LM in patients with CRNENs. As a result, there is limited information available on LM-CRNENs and survival rates. This gap makes it difficult to assess the risk of LM and prognosis for patients with CRNENs. In addition, although some studies have established predictive models, these models primarily focus on imaging diagnosis or (22) other sites (23, 24). There are very few models specifically addressing LM and prognosis in CRNENs. So, identifying the risk and prognostic factors associated with LM in CRNENs and building effective predictive models is imperative for establishing effective early preventive measures and prolonging the survival time of patients.

The aim of this study is to identify the risk factors and prognostic factors for the occurrence of LM in patients with CRNENs and to construct predictive and prognostic nomograms. Compared to previous broad-spectrum nomogram models, our model specifically focused on patients with CRNENs, providing valuable information on the risk factors for LM in CRNENs and the impact on the prognosis of patients. This information can assist clinicians in early interventions and extend the survival time of patients. This study utilized the US SEER database. The results provide a reference for patients with CRNENs and clinicians. Besides, this study developed two web tools to predict the probability of LM and patient prognosis based on the nomogram.

## 2 Methods

### 2.1 Study population

**Figure 1:**
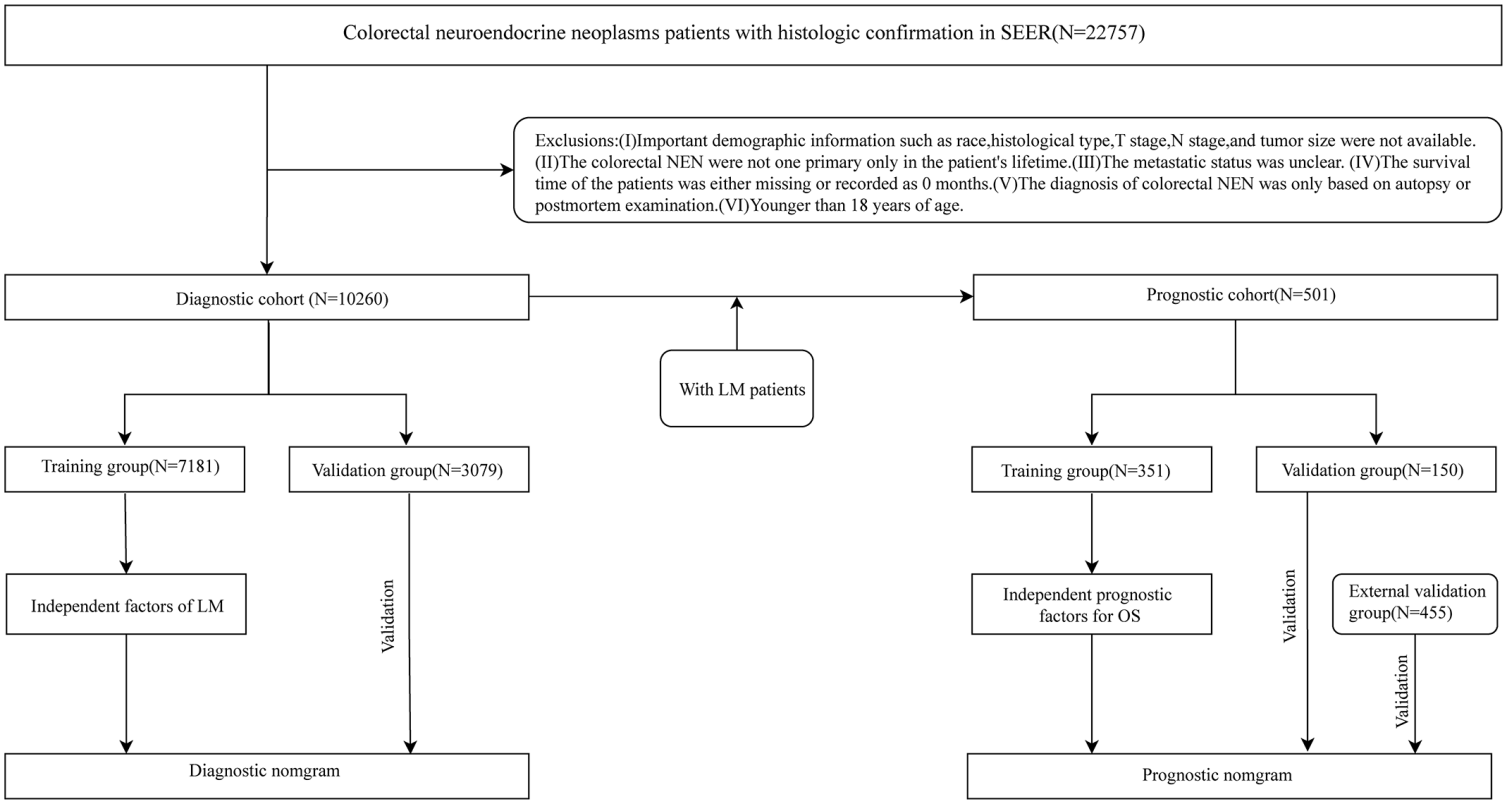
The flow chart of patient selection process.

Our study used the software SEER*Stat 8.4.3 (www.seer.cancer.gov/seerstat) to obtain the clinical information of patients with CRNENs diagnosed by histopathology during 2010-2019 from the SEER database. Patients diagnosed with CRNENs between 2010 and 2019, as recorded in the “Incidence – SEER Research Data, 17 Registries, Nov 2022 Sub (2000-2020)” database, were selected for constructing the risk and prognostic model. Following are the inclusion criteria:1) The primary site of tumors should comply with the International Classification of Diseases for Oncology, 3^rd^ Edition (ICD-O-3): C18.0、C18.2-C18.9、C19.9、C20.9. 2). Histologic type (base on ICD-O-3 codes) (4, 5): 8013/3、8041/3、8043/3、8045/3、8240-8246/3、8249/3、8510/3. 3). Confirmed by histopathology. On the other hand, we formulated some criteria to exclude:1). Clinical and follow-up information is missing, such as Age, T stage, N stage, tumor size, etc. 2). CRNENs are not the patient’s only primary tumor in his/her lifetime. 3). The status of LM is unknown.4). Survival time is 0 months or blacks. 5). Age<18 years. Following the application of the above criteria, a total of 10,260 patients were enrolled in the study. These 10,260 patients formed the predictive cohort. Among these patients, 501 cases developed LM, and they were selected to form the prognostic cohort. Besides, based on the above selection criteria, we collected 445 patients diagnosed with LM between 2010 and 2019 as the external validation group. The patient’s information in the external validation group was from the “Incidence-SEER Research plus Data, 18 Registries, Nov 2022 Sub (2000-2020)” database. The database encompasses more registration centers and covers a more extensive population. This study met the ethical standards of the Helsinki Declaration. **Figure 1** shows the specific selective steps of the study population. C18.0 and 8013/3 et al., respectively, stand for the codes of anatomical location and histologic type within ICD-O-3. For detailed information, please refer to:https://www.who.int/publications/i/item/international-classification-of-diseases-for-oncology.

### 2.2 Variable selection and processing

In this study, we collected fourteen disease-related variables to analyze the risk of LM development in CRNENs. The following are the variables in the predictive cohort: gender, age, marital status, race, site, Histologic type, T stage, N stage, tumor grade, bone metastases, lung metastases, tumor size, surgery, and lymph node surgery. Besides the above variables, chemotherapy and radiotherapy were encompassed in the prognostic cohort. The cutoff value computed by the software X-tile was used to transform continuous variables into dichotomous variables. X-tile identifies the optimal cut-off value for continuous variables by maximizing the discrepancies in survival rates. Based on the optimal cut-off value, continuous variables are converted into categorical variables for analysis (https://aacrjournals.org/clincancerres/article/10/21/7252/183525). Individuals were placed into black, white, and other racial groups. The tumor of primary sites was divided into different parts based on the anatomical site. The site refers to the primary location of the tumor, and surgery indicates that the primary site has been accepted for surgery.

### 2.3 Study methods

This study encompassed two cohorts used to investigate the risk factors and prognostic factors for LM in patients with CRNENs, respectively, and concurrently establish relevant nomogram models. With the intent of evaluating the predictive performance of our model in the target population and clinical environment (25), we processed the two cohorts in the following manner: Firstly, these patients were randomly allocated into a training group (n=7,181) and a validation group (n=3,079) in a 7:3 ratio, to develop a predictive model for LM. The validation group was used to validate the model. Secondly, the 501 patients were divided into a training group (N=351) and an internal validation group (150) at the same ratio to develop a prognostic model. Besides, the external validation group is used to validate the prognostic model. LM was defined as the primary observation endpoint in the predictive cohort. While overall survival (OS) was defined as the primary study endpoint in the prognostic cohort.

### 2.4 Statistical analysis

Quantitative variables were described by mean ± standard deviation (SD), while categorical variables were represented by frequencies and percentages (N %). Statistical analysis and graph plotting were performed using SPSS (26.0) and R (4.3.0) in this study. X-tile was used to calculate cutoff values so as to categorize continuous variables. Chi-square and Wilcoxon rank-sum tests were conducted for intergroup comparisons to ensure balance regarding different baseline information. Univariate logistic regression analysis was used to identify variables related to LM (*p* < 0.05). Subsequently, variables with statistically significant differences (*p* < 0.05) from univariate analysis were included in a multivariate logistic analysis to identify independent factors associated with LM. Finally, the variables associated with LM occurrence were determined. Odds ratios (OR) and 95% confidence intervals (CI) were used to demonstrate the impact of independent factors on LM. In the investigation of prognostic-related indicators, we adopted univariate Cox regression analysis to identify prognostic variables (*p* < 0.05). According to the results of the univariate Cox regression (*p* < 0.05), we used multivariate Cox regression to distinguish independent indicators associated with prognosis. Hazard ratios (HR) and 95% CI were used to demonstrate the role of prognostic indicators for OS. OS was defined as the time from diagnosis to death or the end of follow-up. Employ the lrm and glm functions to fit and conduct hypothesis tests of a logistic model. Fit and perform hypothesis tests by utilizing the Coxph and Cph functions for Cox regression models. *p*<0.05 is considered statistically significant.

### 2.5 Nomogram construction and validation

In our study, two nomograms were established: the LM predictive model for patients with CRNENs and the OS prognostic model for patients with LM-CRNENs. Additionally, based on these two nomograms, we developed two web tools to enhance the practicality of both models. To evaluate the discrimination, calibration, and clinical practicality, the ROC, calibration, and DCA curves were used to evaluate the nomogram (26). Collectively, these evaluation metrics offer a comprehensive assessment of the model’s performance, which is critical for robust model validation. The calibration curve is a scatter plot depicting the actual and predicted event rates. It serves as a visual manifestation of the Hosmer-Lemeshow goodness-of-fit test outcomes. The adopted calibration approach is bootstrapping, and the number of bootstrap iterations is 1000. DCA curve is used to evaluate the net benefit of a predictive model over a range of threshold probabilities, thereby evaluating the clinical utility of the predictive model. Net Benefit $=\frac{TP}{N}-\frac{FP*p}{N}$ . *TP* is the number of true positives, *FP* is the number of false positives, *p* represents the threshold probability (the likelihood at which a clinician would decide to treat a patient), *N* is the total number of patients.

#### 2.5.1 The establishment of the predictive nomogram for LM based on multivariate logistic regression analysis

We constructed the predictive nomogram model according to the results of the multivariable logistic analysis of the training group (N=7181). Following the integration of all independent factors in the nomogram model, it was visualized using R. Subsequently, the model was validated by the validation group (N=3079).

#### 2.5.2 The establishment of the prognostic nomogram for OS based on multivariate Cox regression analysis

The prognostic nomogram model was developed on the results of the multivariable Cox analysis based on the training group (N=351). All independent prognostic factors were incorporated to predict the OS (1-, 2-, and 3-year) of patients with LM-CRNENs. Then, the model was validated internally (N=150) and externally (N=455). The evaluation metrics for the prognostic nomogram were similar to those used for the predictive model, including the ROC, calibration, and DCA curves. Furthermore, each patient’s risk score was calculated by the “TotalPoints.rms” function based on the prognostic nomogram model. Subsequently, we extract the survival time, survival status, and risk score to determine the cutoff values through the X-tile software. Then, the patients were divided into high-risk and low-risk groups based on the cut-off value. Finally, Kaplan-Meier survival analysis and log-rank tests were employed to analyze the differences in OS between the two risk groups. Furthermore, we adjusted for potential confounders.

## 3 Result

### 3.1 Baseline characteristics of patients

Data were collected from the SEER database on 10,260 patients diagnosed with colon or rectal neuroendocrine tumors between 2010 and 2019, including 501 patients with LM. The clinicopathological characteristics of the two groups are shown in **Tables 1** and **2**. Results of the intergroup difference tests indicated no statistical difference in the distribution of variables between the groups (*p*> 0.05).

**Table 1:** Clinicopathological characteristics of patients with CRNENs in predictive cohort.

Characteristics	Total (N=10260)	Training group (N=7181)	Validation group (N=3079)	χ^2^ / |Z|	P-value
**Age**				0.085	0.77
<60	4936 (48.1%)	3462 (48.2%)	1474 (47.9%)		
≥60	5324 (51.9%)	3719 (51.8%)	1605 (52.1%)		
**Sex**				0.688	0.407
Male	4604 (44.9%)	3242 (45.1%)	1362 (44.2%)		
Female	5656 (55.1%)	3939 (54.9%)	1717 (55.8%)		
**Race**				0.663	0.718
Black	1640 (16%)	1144 (15.9%)	496 (16.1%)		
White	7216 (70.3%)	5066 (70.5%)	2150 (69.8%)		
Others	1404 (13.7%)	971 (13.5%)	433 (14.1%)		
**Marital status**				0.474	0.491
Single	4777 (46.6%)	3327 (46.3%)	1450 (47.1%)		
Married	5483 (53.4%)	3854 (53.7%)	1629 (52.9%)		
**Primary Site**				0.262	0.877
Colon	5470 (53.3%)	3828 (53.3%)	1642 (53.3%)		
Rectosigmoid junction	194 (1.9%)	139 (1.9%)	55 (1.8%)		
Rectum	4596 (44.8%)	3214 (44.8%)	1382 (44.9%)		
**Histologic**				12.45	0.087
8013	140 (1.4%)	96 (1.3%)	44 (1.4%)		
8041	96 (0.9%)	60 (0.8%)	36 (1.2%)		
8240	7574 (73.8%)	5320 (74.1%)	2254 (73.2%)		
8244	357 (3.5%)	259 (3.6%)	98 (3.2%)		
8245	80 (0.7%)	58 (0.8%)	22 (0.7%)		
8246	1391 (13.6%)	983 (13.7%)	408 (13.3%)		
8249	173 (1.7%)	108 (1.5%)	65 (2.1%)		
8510	449 (4.4%)	297 (4.1%)	152 (4.9%)		
**T stage**				0.503^*^	0.615
T1	7009 (68.3%)	4892 (68.1%)	2117 (68.8%)		
T2	851 (8.3%)	600 (8.4%)	251 (8.2%)		
T3	1599 (15.6%)	1135 (15.8%)	464 (15.1%)		
T4	801 (7.8%)	554 (7.7%)	247 (8.0%)		
**N stage**				0.456^*^	0.627
N0	8567 (83.5%)	6003 (83.6%)	2564 (83.3%)		
N1	1400 (13.6%)	983 (13.7%)	417 (13.5%)		
N2	293 (2.9%)	195 (2.7%)	98 (3.2%)		
**Grade**				1.096^*^	0.237
I	7812 (76.1%)	5486 (76.4%)	2326 (75.5%)		
II	1001 (9.8%)	703 (9.8%)	298 (9.7%)		
III	1105 (10.8%)	766 (10.7%)	339 (11.0%)		
IV	342 (3.3%)	226 (3.1%)	116 (3.8%)		
**Bone**				0.001	0.976
No	10198 (99.4%)	7137 (99.4%)	3061 (99.4%)		
Yes	62 (0.6%)	44 (0.6%)	18 (0.6%)		
**Liver**				0.046	0.83
No	9759 (95.1%)	6833 (95.2%)	2926 (95.0%)		
Yes	501 (4.9%)	348 (4.8%)	153 (5.0%)		
**Lung**				0.153	0.696
No	10190 (99.3%)	7134 (99.3%)	3056 (99.3%)		
Yes	70 (0.7%)	47 (0.7%)	23 (0.7%)		
**Size(cm)**				0.94	0.332
<1.8	7241 (70.6%)	5089 (70.9%)	2152 (69.9%)		
≥1.8	3019(29.4%)	2092 (29.1%)	927 (30.1%)		
**Surgery**				2.333	0.127
No	527 (5.1%)	385 (5.4%)	142 (4.6%)		
Yes	9733 (94.9%)	6796 (94.6%)	2937 (95.4%)		
**LN Surgery**				0.016	0.900
No	7047 (68.7%)	4929 (68.6%)	2118 (68.8%)		
Yes	3213 (31.3%)	2252 (31.4%)	961 (31.2%)		
**Status**				0.012	0.914
Alive	9040 (88.1%)	6325 (88.1%)	2715 (88.2%)		
Dead	1220 (11.9%)	856 (11.9%)	364 (11.8%)		

The “*” means "The |Z| of the Wilcoxon rank-sum test".

**Table 2:** Clinicopathological characteristics of patients with CRNENs in prognosis cohort.

Characteristics	Total (N=501)	Training group (N=351)	Internal validation group (N=150)	χ^2^ / |Z|	P-value
**Age**				1.094	0.296
<60	240(47.9%)	174 (49.6%)	66 (44.0%)		
≥60	261(52.1%)	177 (50.4%)	84 (56.0%)		
**Sex**				0.633	0.426
Male	229(45.7%)	165 (47.0%)	64 (42.7%)		
Female	272(54.3%)	186 (53.0%)	86 (57.3%)		
**Race**				0.633	0.729
Black	74(14.8%)	49 (14.0%)	25 (16.7%)		
White	395(78.8%)	279 (79.5%)	116 (77.3%)		
Others	32(6.4%)	23 (6.6%)	9 (6.0%)		
**Marital status**				0.669	0.413
Single	205(40.9%)	139 (39.6%)	66 (44.0%)		
Married	296(59.1%)	212 (60.4%)	84 (56.0%)		
**Primary Site**				0.981	0.913
Left	295(58.9%)	208 (59.3%)	87 (58.0%)		
Transverse colon	21(4.2%)	14 (4.0%)	7 (4.7%)		
Right	41(8.2%)	28 (8.0%)	13 (8.7%)		
Rectosigmoid junction	15(3.0%)	12 (3.4%)	3 (2.0%)		
Rectum	129(25.7%)	89 (25.4%)	40 (26.7%)		
**Histologic**				4.877	0.56
8013	53(10.6%)	33 (9.4%)	20 (13.3%)		
8041	43(8.6%)	29 (8.3%)	14 (9.3%)		
8240	116(23.2%)	81 (23.1%)	35 (23.3%)		
8244	26(5.2%)	19 (5.4%)	7 (4.7%)		
8246	227(45.3%)	167 (47.6%)	60 (40.0%)		
8249	21(4.2%)	12 (3.4%)	9 (6.0%)		
8510	15(2.9%)	10 (2.8%)	5 (3.3%)		
**T stage**				0.105^*^	0.917
T1	35(7%)	20 (5.7%)	15 (10.0%)		
T2	75(15%)	51 (14.5%)	24 (16.0%)		
T3	207(41.3%)	147 (41.9%)	60 (40.0%)		
T4	184(36.7%)	133 (37.9%)	51 (34.0%)		
**N stage**				0.212^*^	0.832
N0	106(21.2%)	74 (21.1%)	32 (21.3%)		
N1	317(63.3%)	224 (63.8%)	93 (62.0%)		
N2	78(15.5%)	53 (15.1%)	25 (16.7%)		
**Grade**				1.199^*^	0.230
I	120(24.0%)	81 (23.1%)	39 (26.0%)		
II	60(12.0%)	40 (11.4%)	20 (13.3%)		
III	227(45.3%)	160 (45.6%)	67 (44.7%)		
IV	94(18.7%)	70 (19.9%)	24 (16.0%)		
**Bone**				0	1
No	452(90.2%)	317 (90.3%)	135 (90.0%)		
Yes	49(9.80%)	34 (9.7%)	15 (10.0%)		
**Lung**				0.188	0.664
No	448(89.4%)	312 (88.9%)	136 (90.7%)		
Yes	53(10.6%)	39 (11.1%)	14 (9.3%)		
**Size (cm)**				0.409	0.815
<3.5	160(31.9%)	113 (32.2%)	47 (31.3%)		
3.5-6.5	207(41.3%)	147(41.9%)	60(40.0%)		
≥6.5	134(26.8%)	91(25.9%)	43(28.7%)		
**Surgery**				0.056	0.812
No	135(26.9%)	93 (26.5%)	42 (28.0%)		
Yes	366(73.1%)	258 (73.5%)	108 (72.0%)		
**LN Surgery**				0.931	0.335
No	173(34.5%)	116 (33.0%)	57 (38.0%)		
Yes	328(65.5%)	235 (67.0%)	93 (62.0%)		
**Chemotherapy**				0.287	0.592
No	243(48.5%)	167 (47.6%)	76 (50.7%)		
Yes	258(51.5%)	184 (52.4%)	74 (49.3%)		
**Radiation**				1.694	0.193
No	449(89.6%)	310 (88.3%)	139 (92.7%)		
Yes	52(10.4%)	41 (11.7%)	11 (7.3%)		
**Status**				2.216	0.137
Alive	138 (27.5%)	104 (29.6%)	34 (22.7%)		
Dead	363 (72.5%)	247 (70.4%)	116 (77.3%)		

The “*” means "The |Z| of the Wilcoxon rank-sum test".

Among 10,260 patients with CRNENs, 48.1% of the patients were under the age of 60. whereas 51.9% were above 60 years old. 70.3% were white, 16% were black, and 13.7% were others. The proportion of female patients (55.1%) exceeded that of male patients (44.9%). The incidence in the colon (53.3%) was higher than in the rectum (44.8%) and the rectosigmoid junction (1.9%). The most common histologic type was 8240 (73.8%). At the time of initial diagnosis, over 50% of patients were in T1 (68.3%) and N0 (83.5%) stages, with well-differentiated tumors (GI 76.1%). The liver was the most common site of distant metastasis (4.9%), far more than bone (0.6%) and lung metastases (0.7%). In terms of treatment, 94.9% of patients underwent surgery. Among the 501 patients with LM, those with tumors located in the left colon accounted for 58.9%, and the most common histologic type was 8246 (45.3%). Meanwhile, 9.8% had bone metastasis, and 10.6% had lung metastasis. For treatment, 73.1% of patients underwent surgery, 51.5% received chemotherapy, and 10.4% received radiotherapy. From the baseline characteristics of the predictive cohort, it is notable that the prevalence of CRNENs shows no significant variance across various age groups and genders. The incidence among Caucasians is significantly higher than that of other races. The incidence of the colon is higher than the rectal. Besides demographic characteristics, tumor differentiation and metastasis patterns offer vital insights. Most tumors are located at T0 and N0, with good differentiation, proving that NENs are indolent tumors. Additionally, LM is the most common type, suggesting we should be alert for LM in clinical work. In the prognostic cohort, most of the LM occurred in the left colon, which indicates that we should pay more attention to tumors located in the left colon. Regarding treatment, the ratio of surgery and chemotherapy is significantly higher than that of radiation therapy. This might suggest that patients with LM have a relatively low sensitivity to radiation therapy.

### 3.2 Risk factors and predictive nomogram for LM in CRNENs

#### 3.2.1 Risk factors analysis of LM in CRNENs

In order to find the indicators of LM in CRNENs, we incorporated 14 variables. The results of univariate logistic demonstrate that age, gender, race, histologic type, Grade, T stage, N stage, lung metastasis, bone metastasis, surgery, and tumor size are factors related to the occurrence of LM in patients with CRNENs (**Table 3**; *p* < 0.05). Multivariable logistic analysis results revealed that histologic type, Grade, T stage, N stage, lung metastasis, bone metastasis, surgery, and tumor size are the independent influencing factors for LM in patients with CRNENs (**Table 3**). Besides, the Grade III-IV, T2-T3 stage, N1-N2 stage, bone metastasis, tumor size≥1.8cm, and lung metastasis may serve as risk factors (**Table 3**; OR > 1; *p* < 0.05). In contrast, the histological type (8240;8244;8510) may be the protective indicator (**Table 3**; OR < 1; *p* < 0.05).

**Table 3:** The result of univariate and multivariate logistic analysis.

Characteristics	Univariate analysis			Multivariate analysis		
	OR	CI	P	OR	CI	P
Age						
<60	1.00			1.00		
≥60	2.63	2.06-3.34	<0.001	1.17	0.86-1.61	0.323
Sex						
Male	1.49	1.20-1.85	<0.001	1.21	0.92-1.60	
Female	1.00			1.00		
Race						
Black	0.78	0.57-1.06	0.111	1.30	0.86-1.93	0.207
White	1.00			1.00		
Others	0.42	0.27-0.65	<0.001	0.80	0.44 - 1.39	0.442
Marital status						
Single	1.00			1.00		
Married	1.27	1.02-1.58	0.034	1.25	0.94 - 1.67	0.120
Primary Site						
Colon	1.00			1.00		
Rectosigmoid junction	1.27	0.68-2.38	0.46	1.49	0.58 - 3.55	0.378
Rectum	0.44	0.35-0.57	<0.001	0.73	0.46 - 1.15	0.180
Histologic						
8013	1.00			1.00		
8041	1.27	0.66-2.46	0.469	0.84	0.38 – 1.85	0.666
8240	0.03	0.02-0.04	<0.001	0.51	0.27 - 0.97	0.040
8244	0.14	0.08-0.26	<0.001	0.20	0.10 - 0.40	<0.001
8245	0.03	0.00-0.22	0.001	0.13	0.01 - 0.78	0.067
8246	0.31	0.20-0.49	<0.001	0.91	0.53 - 1.59	0.736
8249	0.27	0.14-0.53	<0.001	1.45	0.56 - 3.61	0.434
8510	0.06	0.03-0.13	<0.001	0.09	0.04 - 0.20	<0.001
Grade						
I	1.00			1.00		
II	3.81	2.57-5.65	<0.001	1.30	0.8 - 2.08	0.278
III	17.90	13.53-23.67	<0.001	2.60	1.66 - 4.09	<0.001
IV	28.12	19.62-40.30	<0.001	3.38	1.95 - 5.86	<0.001
T stage						
T1	1.00			1.00		
T2	19.65	12.14-31.78	<0.001	2.70	1.4 - 5.25	0.003
T3	29.19	19.00-44.84	<0.001	3.86	2.03 - 7.47	<0.001
T4	53.83	34.6-83.74	<0.001	4.46	2.26 - 8.98	<0.001
N stage						
N0	1.00			1.00		
N1	23.65	17.99-31.08	<0.001	3.91	2.65 - 5.87	<0.001
N2	27.63	18.62-41.00	<0.001	4.11	2.35 - 7.25	<0.001
Bone						
No	1.00			1.00		
Yes	73.88	36.17-150.89	<0.001	7.75	3.01 - 21.14	<0.001
Lung						
No	1.00			1.00		
Yes	92.94	44.55-193.92	<0.001	8.91	3.55 - 24.72	<0.001
Size (cm)						
<1.8	1.00			1.00		
≥1.8	33.98	22.86-50.52	<0.001	3.32	1.92 - 5.93	<0.001
Surgery						
No	1.00			1.00		
Yes	0.11	0.08-0.14	<0.001	0.17	0.1 - 0.31	<0.001
LN Surgery						
No	1.00			1.00		
Yes	4.17	3.33-5.22	<0.001	0.78	0.44 - 1.42	0.41

#### 3.2.2 The predictive nomogram for LM in CRNENs

**Figure 2:**
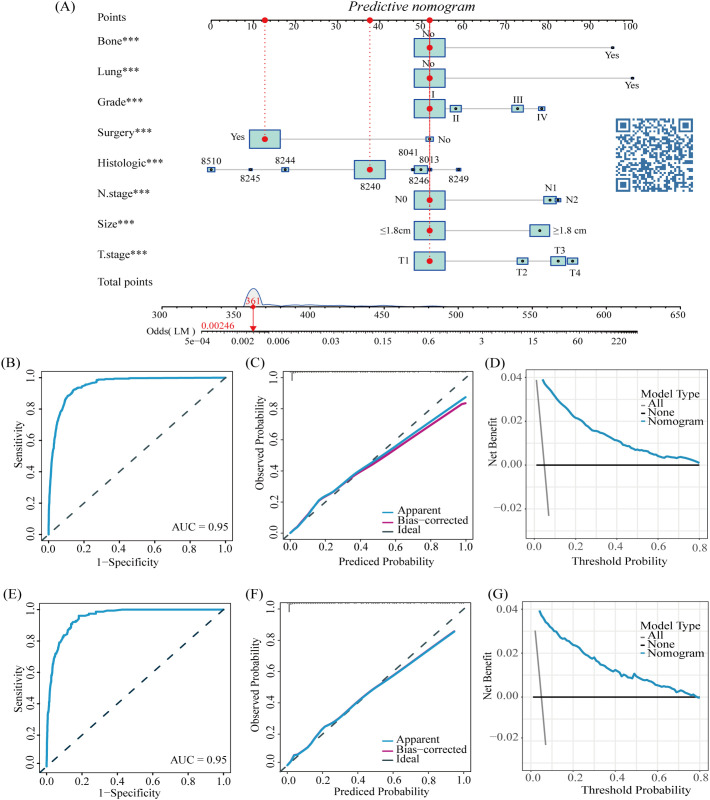
The nomogram for LM in patients with CRNENs **(A)**, and the ROC curves for the predictive nomogram in the training **(B)** and validation group **(E)** Calibration curves in the training **(C)** and validation group **(F)** DCA curves in the training **(D)** and validation group **(G)**. The "*" indicates the significance level of the independent variable. The number of "*" varies when the significance levels are different.

**Figure 3:**
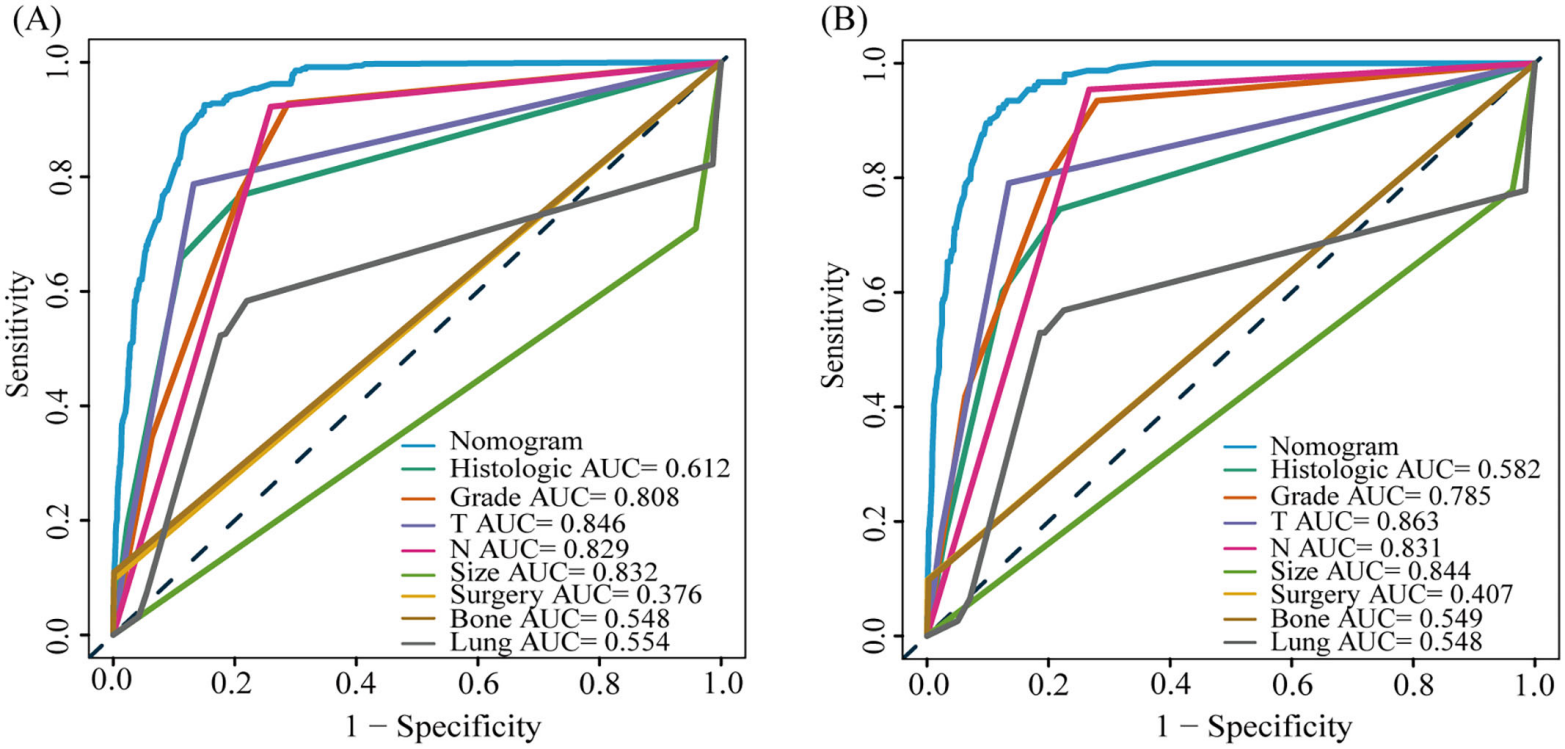
The AUC of predictive nomogram and all factors in the training **(A)** and validation group **(B)**.

According to all independent indicators, we developed a predictive nomogram to predict the possibility of LM in each patient with CRNENs (**Figure 2A**). Subsequently, we evaluated the accuracy of the nomogram. The model demonstrated a strong discriminative ability with an area under curve (AUC) of 0.95 in both the training (**Figure 2B**) and validation groups (**Figure 2E**). This indicates that the model is capable of predicting patients with a high probability of LM at an early stage, facilitating early detection and formulation of treatment plans. Additionally, we plotted calibration curves to assess the predictive accuracy of the nomogram. Both in the training (**Figure 2C**) and validation (**Figure 2F**) groups, the nomogram prediction curve closely aligns with the calibration curve, indicating a high level of prediction accuracy. Meanwhile, the DCA curves illustrated the high clinical utility of the model (**Figures 2D, G**). Furthermore, ROC curves were constructed for each independent factor. The results revealed that the AUC of our composite predictive model exceeded that of individual factors in both the training (**Figure 3A**) and validation (**Figure 3B**) groups. That further demonstrated the superior predictive capacity of our nomogram model for LM compared to single factors.

### 3.3 Prognostic factors and nomogram for LM-CRNENs

#### 3.3.1 Prognostic factors analysis of LM-CRNENs

Among the 10,260 patients with CRNENs, 501 patients with LM, as outlined in **Table 2**. The univariate Cox analysis identified significant factors influencing the prognosis of patients with LM-CRNENs, including race, age, tumor grade, site, histological type, N stage, bone metastasis, lung metastasis, surgery, tumor size, and chemotherapy (**Table 4**; *p* < 0.05). Furthermore, the multivariable Cox analysis revealed that histologic type, age, site, Grade, N stage, tumor size, surgery, and chemotherapy were independent prognostic indicators. Advanced age (≥ 60 years), higher tumor grades (III and IV), N1 stage, and tumor size ≥ 3.5 cm were considered as prognostic adverse determinants (**Table 4**; HR > 1, *p* < 0.05). Primary tumors originating in the rectum, histologic types 8240 and 8510, surgery, and chemotherapy were considered protective factors in prognosis (**Table 4**; HR < 1, *p*<0.05).

**Table 4:** The result of univariate and multivariate COX regression analysis.

Characteristics	Univariate analysis			Multivariate analysis		
	HR	CI	P	HR	CI	P
Age						
<60	1			1		
≥60	1.33	1.04 - 1.72	0.024	1.33	1.01 - 1.74	0.041
Sex						
Male	1.12	0.87 - 1.44	0.381	**—**	**—**	
Female	1					
Race						
Black	0.94	0.66 - 1.35	0.749	0.96	0.65 - 1.43	
White	1			1		
Others	0.58	0.33 - 0.99	0.047	0.69	0.39 - 1.25	0.223
Marital status						
Single	1					
Married	0.85	0.66 - 1.09	0.195	**—**	**—**	**—**
Primary Site						
Left	1					
Transverse colon	1.95	1.1 - 3.45	0.022	1.6	0.88 - 2.92	0.123
Right	2.27	1.45 - 3.58	<0.001	1.05	0.64 - 1.72	0.844
Rectosigmoid junction	0.95	0.49 - 1.87	0.891	0.60	0.29 – 1.21	0.154
Rectum	0.92	0.68 - 1.24	0.575	0.66	0.44 - 0.98	0.040
Histologic						
8013	1			1		
8041	1.12	0.67 - 1.87	0.673	0.87	0.50 - 1.53	0.640
8240	0.17	0.1 - 0.28	<0.001	0.46	0.25 - 0.86	0.015
8244	0.99	0.55 - 1.8	0.985	1.11	0.60 - 2.07	0.733
8246	0.58	0.39 - 0.87	0.008	0.76	0.48 - 1.21	0.243
8249	0.24	0.09 - 0.69	0.008	0.68	0.21 - 2.26	0.530
8510	0.34	0.14 - 0.82	0.016	0.20	0.08 - 0.51	<0.001
Grade						
I	1			1		
II	1.23	0.66 - 2.27	0.516	1.01	0.52 – 1.95	0.975
III	6.5	4.29 - 9.85	<0.001	5.94	3.46 – 10.17	<0.001
IV	5.39	3.43 - 8.48	<0.001	3.92	2.19 - 7.04	<0.001
T stage						
T1	1					
T2	0.91	0.47 – 1.76	0.775	**—**	**—**	**—**
T3	1.02	0.56 – 1.86	0.942	**—**	**—**	**—**
T4	1.53	0.84-2.78	0.165	**—**	**—**	**—**
N stage						
N0	1			1		
N1	1.68	1.19 - 2.38	0.003	1.47	1.00 - 2.15	0.049
N2	3.01	1.95 - 4.64	<0.001	1.47	0.85 - 2.54	0.163
Bone						
No	1			1		
Yes	1.63	1.11 - 2.39	0.013	1.42	0.91 - 2.21	0.124
Lung						
No	1			1		
Yes	1.57	1.08 - 2.28	0.018	1.14	0.76 - 1.70	0.527
Size (cm)						
<3.5	1			1		
3.5-6.5	2.32	1.79 - 3.01	<0.001	1.70	1.19 - 2.41	0.003
≥6.9	2.32	1.79 - 3.01	<0.001	2.44	1.63 – 3.65	<0.001
Surgery						
No	1			1		
Yes	0.67	0.51 - 0.88	0.004	0.45	0.31 - 0.67	<0.001
LN Surgery						
No	1					
Yes	0.89	0.68 - 1.16	0.39	**—**	**—**	**—**
Radiation						
No	1					
Yes	0.99	0.67 - 1.46	0.949	**—**	**—**	**—**
Chemotherapy						
No	1			1		
Yes	1.61	1.24 - 2.08	<0.001	0.50	0.37 - 0.69	<0.001

#### 3.3.2 The prognostic nomogram for LM-CRNENs

**Figure 4:**
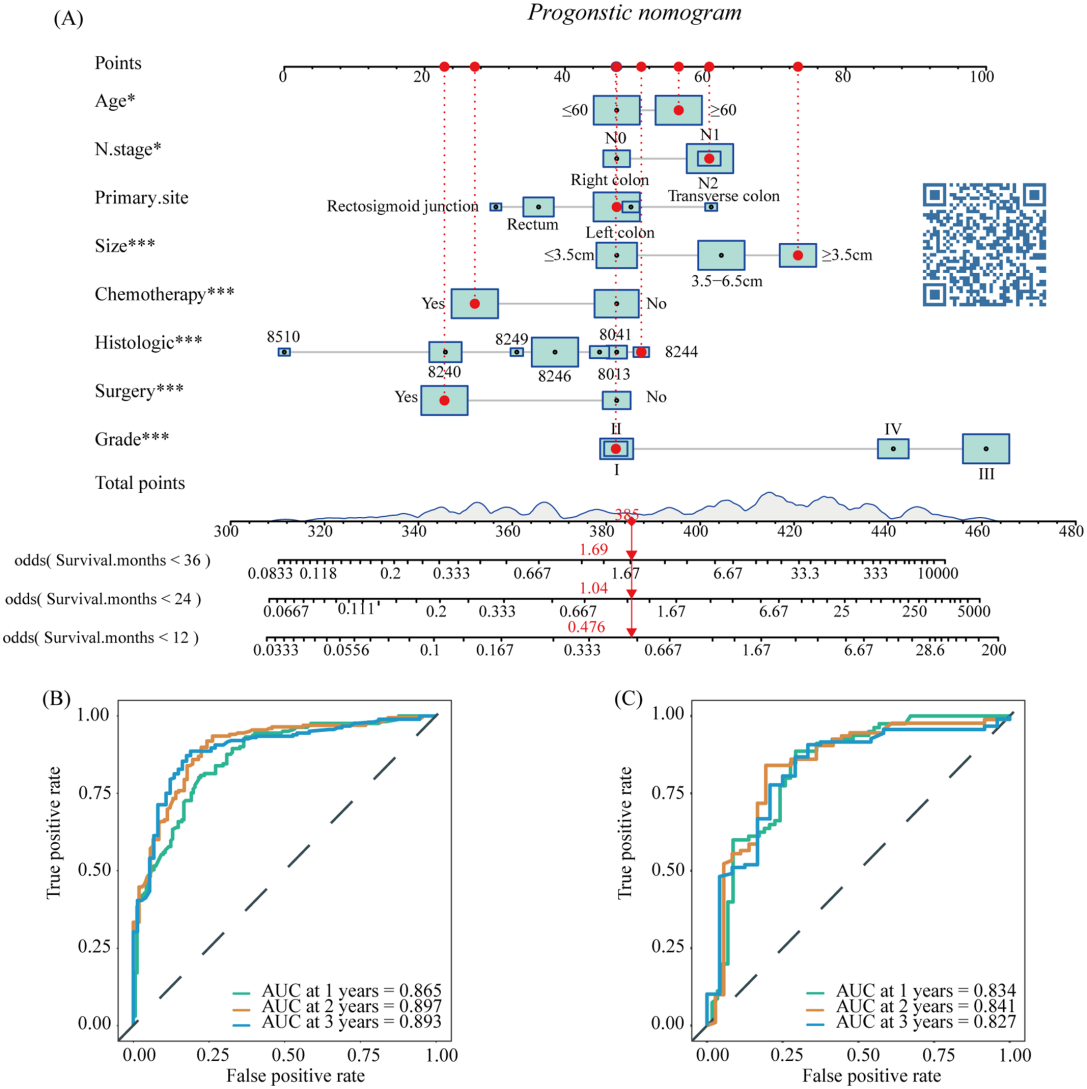
The nomogram survival prediction model for LM in patients with CRNENs **(A)**, and the ROC curve of the prognostic nomogram for 1-year, 2-year, and 3-year in the training **(B)** and the internal validation group **(C)**. The "*" indicates the significance level of the independent variable. The number of "*" varies when the significance levels are different.

**Figure 5:**
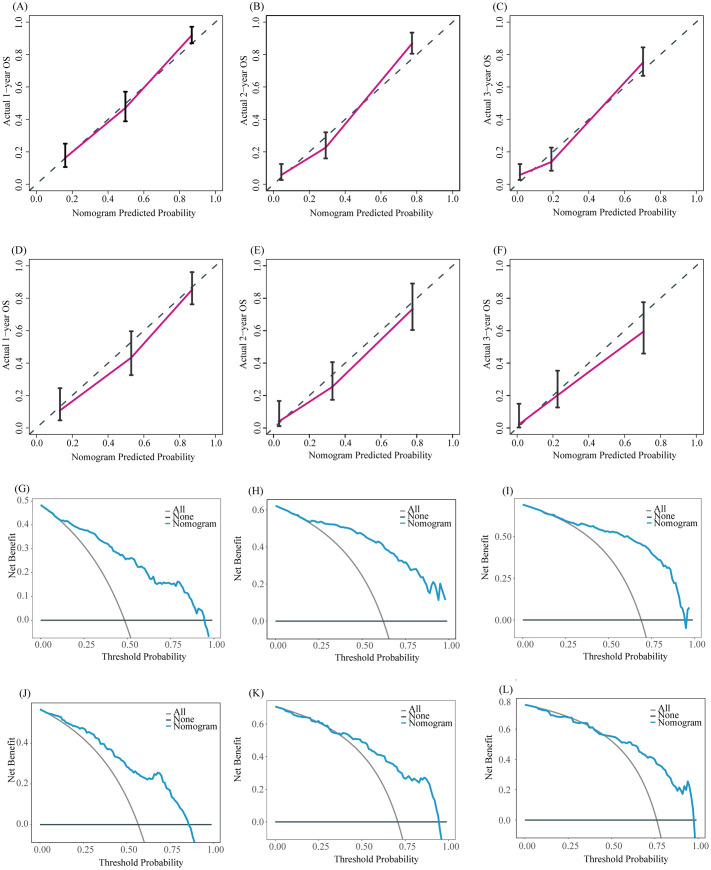
The calibration curves of the prognostic nomogram for the 1-year, 2-year, and 3-year in the training **(A–C)** and internal validation group **(D–F)**. DCA curves for the 1-year, 2-year, and 3-year prognostic nomograms in the training **(G–I)** and internal validation group **(J–L)**.

**Figure 6:**
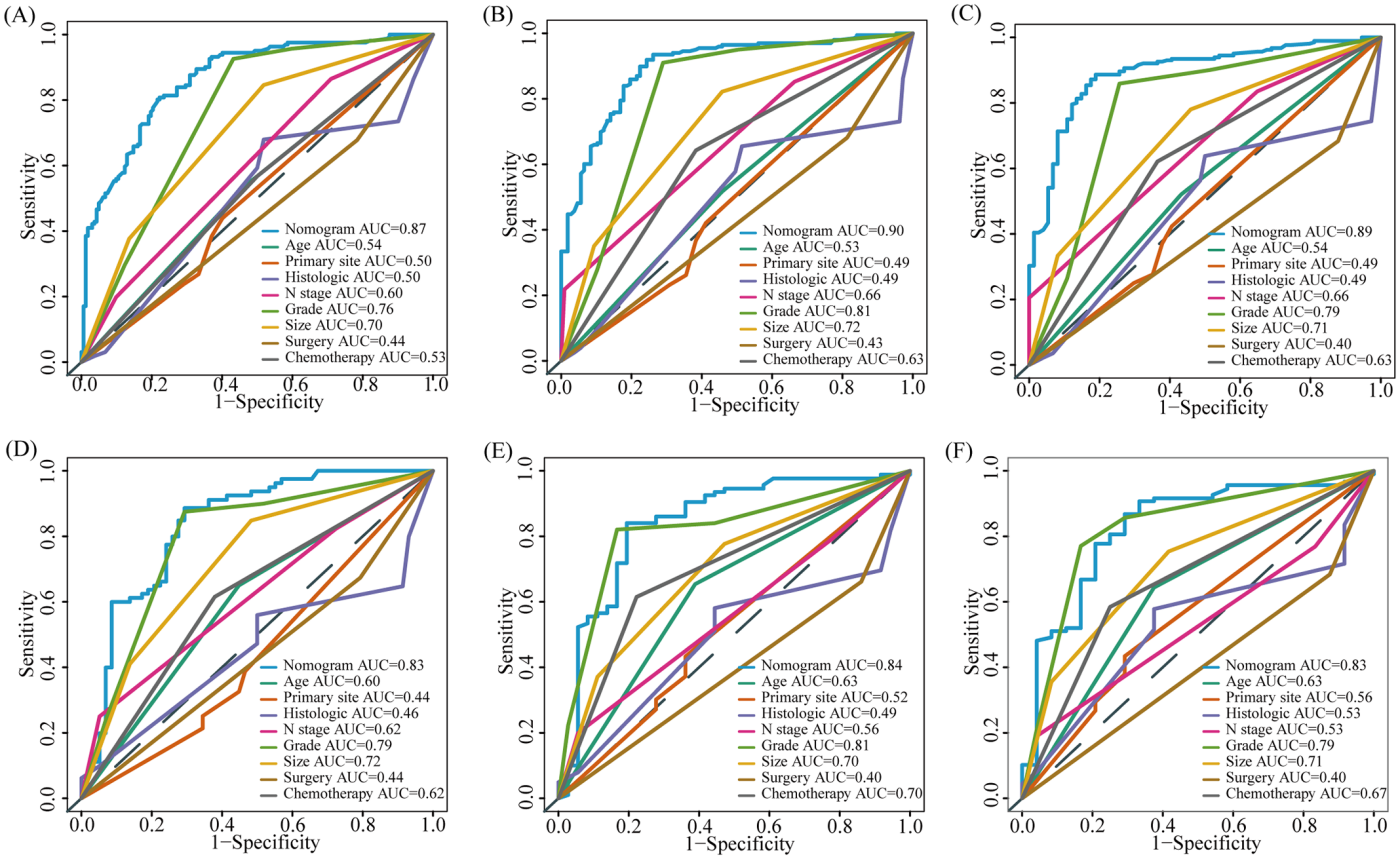
The AUC of prognostic nomogram and all factors in the training **(A–C)** and internal validation group **(D–F)**.

Based on the results of multivariate COX analysis, we constructed a prognostic nomogram to predict the survival rates of patients with LM-CRNENs at 1, 2, and 3 years (**Figure 4A**). Then, we evaluated the nomogram model. The AUC for 1-, 2-, and 3-year OS of the nomogram in the training group were 0.865, 0.897, and 0.893 (**Figure 4B**, *p* < 0.05), and those in the internal validation group were 0.834, 0.841, and 0.827, respectively (**Figure 4C**, *p* < 0.05). In both the training (**Figures 5A–C**) and internal validation groups (**Figures 5D–F**), calibration curves showed that the predicted survival at 1-, 2-, and 3-years was highly consistent with the actual survival, indicating the high concordance and accuracy of our nomogram model. DCA curves also demonstrated that our constructed nomogram model could effectively predict the 1-, 2-, and 3-year OS of patients with LM-CRNENs in clinical practice, whether it is the training group (**Figures 5G–I**) or the internal validation group (**Figures 5J–L**).In addition, in the training group (**Figures 6A–C**, *p* < 0.05) and internal validation group (**Figures 6D–F**, *p* < 0.05), we plotted ROC curves for the prediction of OS at different years based on eight independent factors and compared their AUC values with our nomogram model. The results indicated that the predictive ability of the nomogram model for OS at different years was higher than that of individual factors both in the training and internal validation group.

#### 3.3.3 External validation of prognostic nomogram

**Figure 7:**
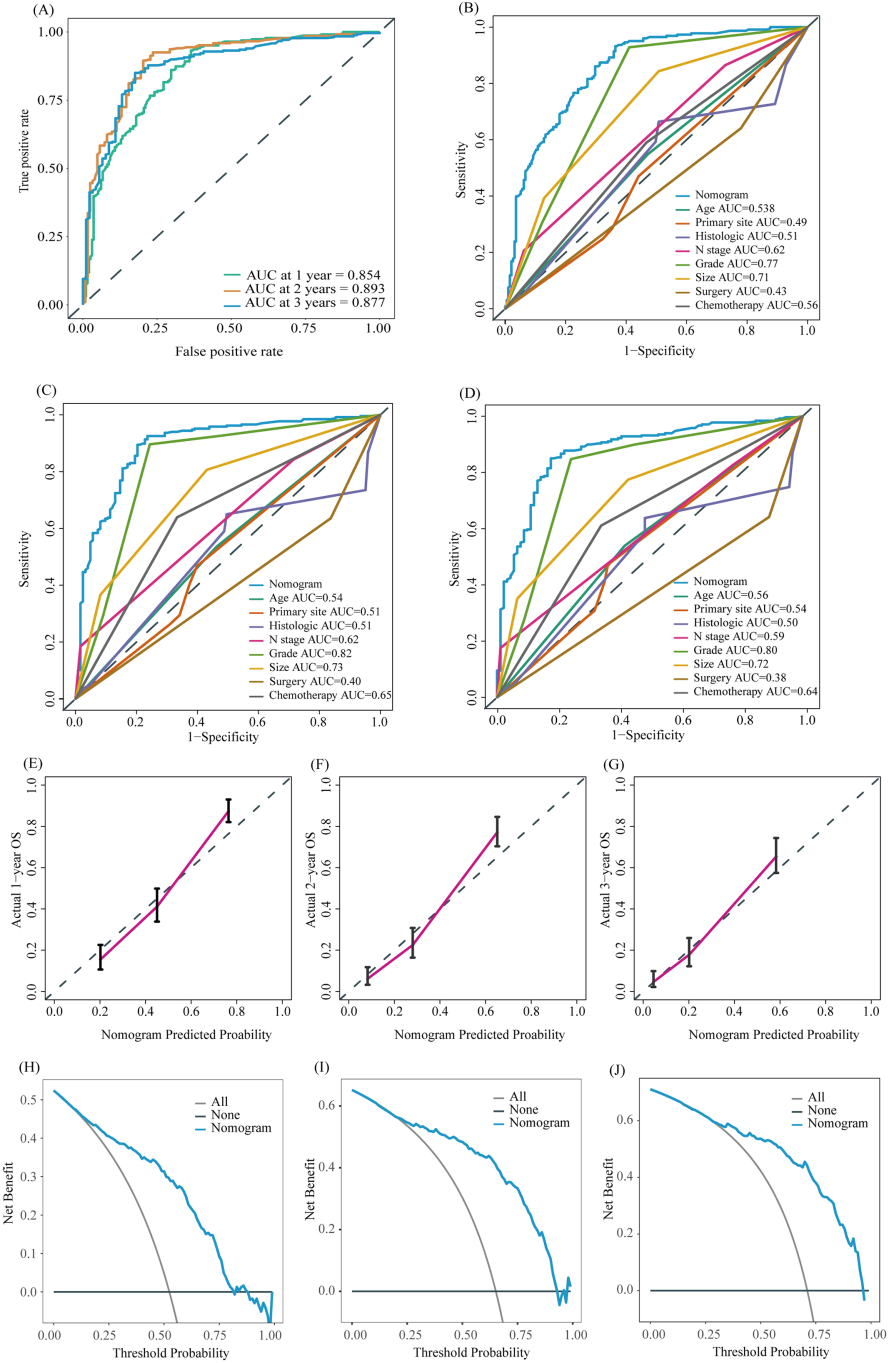
ROC curve of the prognostic nomogram for 1-year, 2-year, and 3-year in the external validation group **(A)**. The AUC between prognostic nomogram and all factors in the external validation group **(B–D)** of 1-, 2-, 3-years. Calibration curves of the prognostic nomogram for the 1-year, 2-year, and 3-year in the external validation group **(E–G)** The DCA curves of the prognostic nomogram for the 1-year, 2-year, and 3-year in the external validation group **(H–J)**.

To further evaluate the predictive capability of the prognostic nomogram model, we conducted validation using an external validation group. The external validation group was composed of a population not involved in model development. Although it is demographic characteristics and inclusion criteria are similar to those of the training/internal validation group, this cohort represents a population from a wider geographic area. Validating the model using this cohort further evaluates its generalization ability and enhances its credibility and practicality. The model also performed well in the external validation group. The AUC of 1-, 2-, and 3-year OS of our nomogram model in the external validation group were 0.854, 0.893, and 0.877, respectively (**Figure 7A**, *p* < 0.05), while the AUC of individual factors were lower (**Figures 7B–D**, *p* < 0.05). The calibration curves of the 1-, 2-, and 3-year OS probability nomograms demonstrated that the predicted results were highly consistent with the actual outcomes (**Figures 7E–G**). The DCA curves also have a good performance (**Figures 7H–J**).

**Figure 8:**
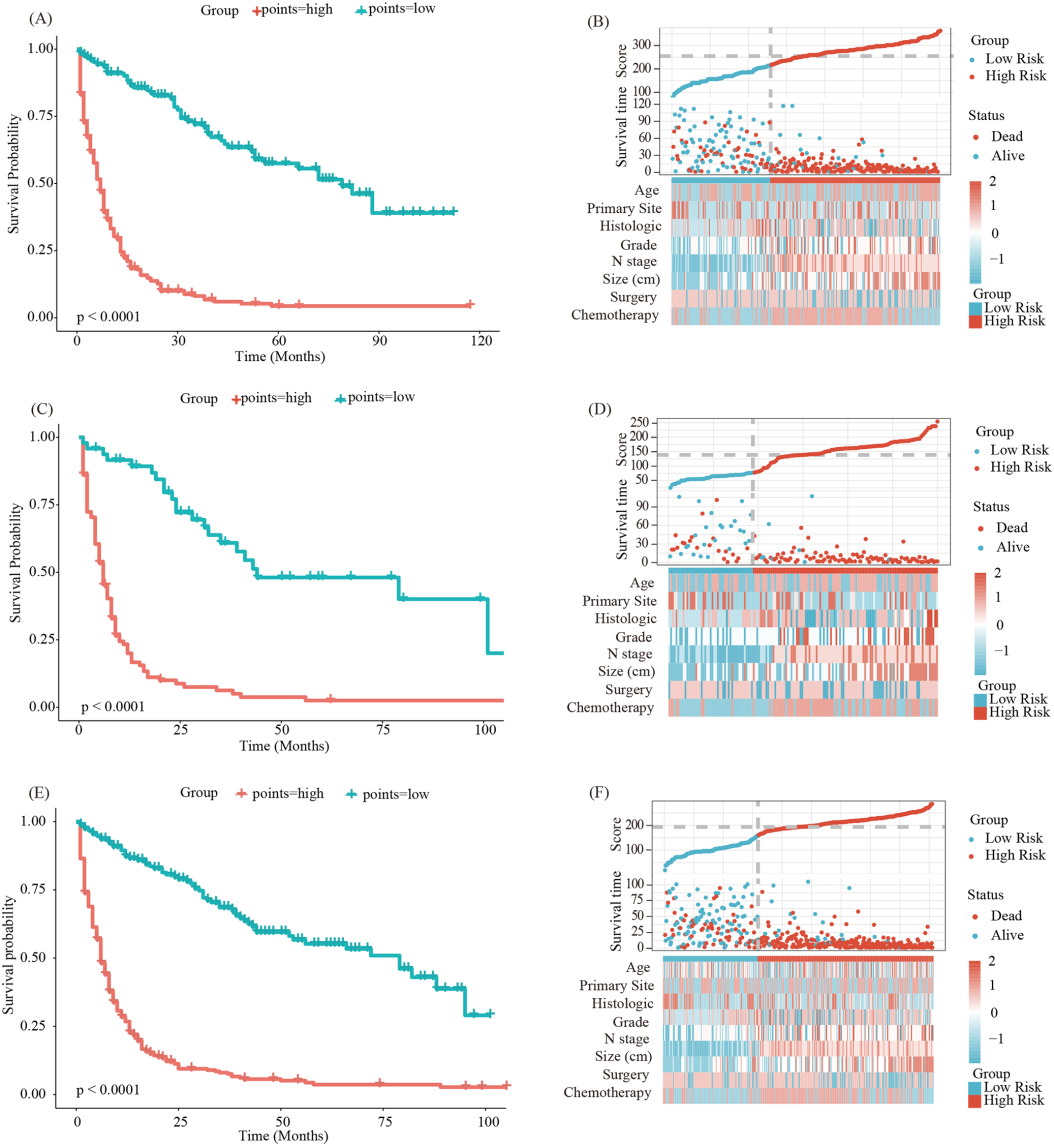
Survival curves and the distribution of clinicopathological features in different groups for OS in the training group **(A, B)**, validation group **(C, D)** and external validation group **(E, F)**.

Finally, we utilized the model to calculate scores for each patient in the training, internal validation, and external validation group, identifying optimal cutoff values for each group to divide patients into high-risk and low-risk groups for Kaplan-Meier survival analysis. Kaplan-Meier survival analysis indicated that the prognosis for patients in the high-risk group was significantly poorer compared to those in the low-risk group across the training group (**Figures 8A, B**, *p* < 0.05), internal validation group (**Figures 8C, D**, *p* < 0.05), and external validation group (**Figures 8E, F**, *p* < 0.05). In summary, the nomogram model demonstrated good performance in predicting the OS of patients with LM-CRNENs.

## 4 Discussion

NENs show significant heterogeneity in their clinical behavior, histological features, molecular characteristics, and response to treatment. The heterogeneity can manifest as differences in tumor grade, tumor location, hormone production, and overall prognosis. However, these mechanisms of heterogeneity remain unclear. They may be related to genetic variations (27), the tumor microenvironment (28), and epigenetic modifications (29), among other factors. The global incidence of NENs has recently continuously increased and has become a serious threat to human health (3, 5, 6). Previous studies have shown that over 50% of patients with NEN already have distant metastases at first diagnosis (15, 16, 30). The liver is the most frequent location of distant metastasis (31). The patient’s survival time will decrease significantly when LM occurs (16, 19). Surgery is the primary treatment for early-stage NENs, which can prolong patient survival time (32). For advanced CRNENs, treatment methods mainly include long-acting somatostatin analogs (SSAs), molecular targeted therapy (TT), chemotherapy (Che), and peptide receptor radionuclide therapy (PRRT). Although those treatments can somewhat improve patient survival time, there are still some issues. For example, extended use of SSAs may lead to drug resistance (33), TT can cause hypertension or bleeding (34), and the side effects of Che, especially hair loss, are unacceptable to most people (35). However, PRRT is not yet globally accessible, and introducing radioactive matter into the body might affect the health of nearby people (34). Therefore, it is significant to identify the risk factors for LM in patients with CRNENs, develop effective prevention measures, and improve patient prognosis. However, there are few available studies on LM and the prognosis of CRNENs, which makes it difficult to assess the risk factors for LM and the prognostic factors of CRNENs. It is crucial to identify those factors that can assist clinicians in developing timely interventions to improve patient survival time.

Nomograms originated in 1884 and were initially used in engineering. Because of its ability to visually represent complex calculations rapidly, intuitively, and precisely, the nomogram has been increasingly used in clinical decision-making and research in the medical field (36). Some studies show that the nomogram has shown good performance in predicting survival outcomes in various types of tumors, such as lung cancer (37), liver cancer (38), gliomas (39), etc. Additionally, it has demonstrated excellent performance in predicting metastasis risk (40), treatment efficacy (41), and tumor biology behavior (42). This indicates that the nomogram is widely used as an important auxiliary tool for clinicians in devising treatment plans.

The T and N stages in the TNM staging system are recognized as crucial factors in the development of distant metastasis in tumors, and higher T and N stages are correlated with an increased propensity for distant metastasis (43, 44). Furthermore, the degree of differentiation is also considered a key determinant of metastatic potential (45). Within the SEER database, the differentiation is stratified based on the Ki-67 index and mitosis into well-differentiated (GI), moderately differentiated (GII), poorly differentiated (G III), and undifferentiated (G IV) categories. Research suggests that the lower the degree of differentiation, the higher the probability of metastasis (45). In this study, our results also support the point that the advanced T stage (OR: T2 vs T3 vs T4 = 2.70 vs 3.86 vs 4.46;*p*<0.05), N stage (OR: N1 vs N2 = 3.91 vs 4.11; *p*<0.05), and poor differentiation (OR: G III vs G IV = 2.6 vs 3.38;*p*<0.05) have a positive correlation with the occurrence of LM in patients with CRNENs. This might be attributed to higher T and N staging and poorly differentiated tumors, which are more aggressive and more accessible to LM. This phenomenon is also consonant with clinical experience. Additionally, tumor size is considered an important indicator of distant metastasis. Previous research has indicated that when the tumor size is ≥1.15 cm, the probability of distant metastasis in CRNENs significantly rises (31). Consistent with this evidence, Our study shows that when tumor diameter is ≥ 1.8 cm (OR: 3.32; *p*<0.05), the likelihood of LM increases. This may be associated with the depth of tumor infiltration and the colorectal venous reflux system (46). Patients with distant metastasis in other sites have a higher possibility of occurring LM. Previous studies have shown that some patients already have lung metastasis combined with LM at the time of diagnosis, and the frequency increases over time (47). Additionally, concomitant lung and bone metastasis patients are more likely to develop LM (47). Another study indicated that patients with lung or LM are significantly more predisposed to developing bone metastasis compared to those without lung or LM (19). Consistent with those studies, according to our study results, out of the 70 patients with lung metastasis, 53 (76%) had LM simultaneously. Meanwhile, among the 62 patients with bone metastasis, 49 (79%) cases were found to have LM. This may be related to the liver’s hemodynamic circulation and tumors’ immune evasion mechanisms. The underlying mechanisms require further investigation. Furthermore, our study analyzed the impact of surgery and histologic types on LM. As shown in the results, surgery may be a protective factor of LM, consistent with previous research (48), possibly due to early detection and timely surgical intervention. Additionally, ICD-O-3 codes 8240, 8244, and 8510 may also act as protective factors against the occurrence of LM, However, this finding has not been reported in previous studies and may require verification with a larger sample size.

The liver is the most common organ of distant metastasis for GEP-NENs. Similarly, this situation also exists in CRNENs. Xu et al. found that rectal neuroendocrine tumors had the best prognosis before metastasis. In contrast, the median OS for metastatic rectal neuroendocrine was only 9-11 months. As Consistent with previous research (19, 49), our study indicates that the following factors will make the patients have a poorer OS for LM-CRNENs, including age ≥ 60, poor tumor differentiation (Grade III-IV), higher N stage, bone metastases, size ≥ 3.5 cm. Older patients may have additional health conditions impacting their ability to tolerate treatment and overall survival. Tumor size is directly associated with tumor burden and the likelihood of local invasion, metastasis, and the efficacy of surgical resection (31). In larger tumors, the challenges of complete surgical removal increase, which can impact recurrence rates and overall prognosis. Poor differentiation, higher N stage, and bone metastases increase the tumor burden, which makes the prognosis of the patient worse. For patients with these factors, timely implementation of alternative treatments may be necessary to improve their prognosis. In terms of treatment, radiotherapy and chemotherapy have consistently been the standard therapeutic approaches for patients with unresectable primary tumors. Consistent with previous studies (31, 50), our results showed that chemotherapy can prolong OS, while radiotherapy does not significantly prolong the OS of patients with LM-CRNENs. The following are some possible reasons. Firstly, radiotherapy is generally believed to be beneficial in reducing local recurrence but does not improve survival rates (51). Secondly, some inevitable confounding factors exist, and the population is relatively limited. Thirdly, the SEER database does not provide specific radiotherapy regimens, which could also be a contributing factor. There are still some debates in the treatment of advanced tumors. For previous studies, palliative surgery is always applicable to patients who have obstructions, bleeding, or perforations due to tumors and is not beneficial for patients with distant metastasis (52, 53). However, recent studies suggest that palliative surgery can reduce tumor burden and improve patient survival time (54). Moreover, it is recommended that simultaneous surgical treatment be applied to patients with LM to enhance long-term survival (50). Those are consistent with our results. Furthermore, in our study, we further analyzed the impact of different locations and histologic types on prognosis, and the results indicated that tumors located in the rectum may contribute to a longer OS in patients with LM. It is also consistent with previous research findings (55). Among different histologic types, 8240 and 8510 may have better OS. there is currently no related research. It might be associated with molecular or genetic changes, which need to be confirmed by further studies.

In our study, eligible patients were included to analyze the factors of LM and prognostic factors of patients with LM-CRNENs. Subsequently, two nomograms were constructed to predict these patients’ risk of LM and prognosis. Based on the validation results, our model shows excellent predictive performance in both the internal and external validation groups. Therefore, this nomogram model can serve as a predictive tool for the occurrence of LM and survival rates in patients with CRNENs. Based on the predictive outcomes, targeted interventions and treatment plans can be implemented to improve patient survival time. Several studies have already established nomogram models related to GEP-NENs. For instance, Xinwei Li et al (56). created a nomogram for predicting distant metastasis in GEP-NENs using data from the SEER database. Adrienne B. Shannon et al (57). also developed a nomogram predictive model for lymph node metastasis in stage I-III non-functional GEP-NENs. Compared to existing ones, our model focuses specifically on patients with CRNENs. By analyzing relevant clinical factors of CRNENs, we have constructed predictive models for LM and prognosis to provide a more precise and systematic study of the risks and outcomes for patients with LM-CRNENs. In addition, we developed two web tools based on the nomograms. These tools can predict the probability of LM and prognosis for patients. When relevant factors are inputted into the online tools, the probability of metastasis and prognosis can be acquired, which can offer decision-making information for clinicians.

In addition, this study still has several limitations. Firstly, although we validated the established model using internal and external data, these patients’ information was sourced from SEER databases and lacked our own clinical follow-up data for validation. Consequently, in future studies, we plan to collect more data to enhance the accuracy of the model. Additionally, the SEER database lacks information on important prognostic biomarkers such as Chromogranin A (CgA), Synaptophysin (Syn), and CD56 (58) et al. Besides, many tumors have genetic mutations, but no corresponding indicator exists in the SEER database. Some studies indicate that KRAS, TP53, ALP (alkaline phosphatase), et al. strongly relate to the response to treatment and prognosis (59). These findings further emphasize the necessity for integrating biomarker features into prognostic models. In future research, we intend to incorporate these biomarkers and some genes through deep sequencing, immunohistochemistry and other fundamental experiments to improve the comprehensiveness and utility of our model. Lastly, because the specific information on radiotherapy and chemotherapy was not provided in the SEER database, it affects the formulation of treatment plans for improving prognosis. In future studies, we will gather more detailed treatment information and further analyze it to identify effective treatment options. Additionally, since the SEER data is based on a sample of the US population and does not cover all regions and ethnic groups, our analysis results may have some bias, indicating the need for further large-scale prospective studies to confirm our findings. Although our nomogram model seemingly exhibits high performance, its applicability might be restricted in diverse patient cohorts or distinct medical settings. Therefore, validation in multi-center cohorts and varying demographics is essential to ensure these models hold across different patient populations. In future research, we intend to incorporate additional variables, such as biomarkers, and employ machine learning algorithms to establish more accurate models.

## 5 Conclusion

Over the past 20 years, although the number of patients with CRNENs has gradually increased, the advancement in treatment methods has led to a continuous improvement in survival time. Histological type, tumor grade, T stage, N stage, bone and lung metastases, as well as tumor size are considered to be associated with the occurrence of LM. Factors influencing overall survival (OS) include age, primary tumor site, histological type, tumor grade, N stage, presence of bone metastases, primary site surgery, tumor size, and chemotherapy. The model we have constructed can accurately predict the probability of LM and has a high predictive performance for OS prognosis. However, there are still many challenges in its future clinical application, and further research should focus on translating the model into practical clinical use.

## Data Availability

Publicly available datasets were analyzed in this study. This data can be found here: http://seer.cancer.gov/seerstat. Contact the corresponding author for access if necessary.
