# Corrigendum: Predictive and prognostic nomogram models for liver metastasis in colorectal neuroendocrine neoplasms: a large population study

**DOI:** 10.3389/fendo.2025.1591823

**Published:** 2025-04-28

**Authors:** Xiao Lei, Yanwei Su, Rui Lei, Dongyang Zhang, Zimeng Liu, Xiangke Li, Minjie Yang, Jiaxin Pei, Yanyan Chi, Lijie Song

**Affiliations:** ^1^ Department of Oncology, The First Affiliated Hospital of Zhengzhou University, Zhengzhou, China; ^2^ Henan Neuroendocrine Tumor Medical Center, The First Affiliated Hospital of Zhengzhou University, Zhengzhou, China; ^3^ Department of Endocrinology, Zhoukou First People’s Hospital, Zhoukou, China; ^4^ School of Basic Medical Sciences, Xinxiang Medical University, Xinxiang, China

**Keywords:** colorectal neuroendocrine neoplasms, liver metastases, overall survival, nomogram, SEER, prognostic factors, risk factors

In the published article, there was an error in the legend of **Figure 2A** as published. Due to the change of the website name, the QR code in the original picture has become invalid. The corrected figure and its caption appear below.

**Figure 2:**
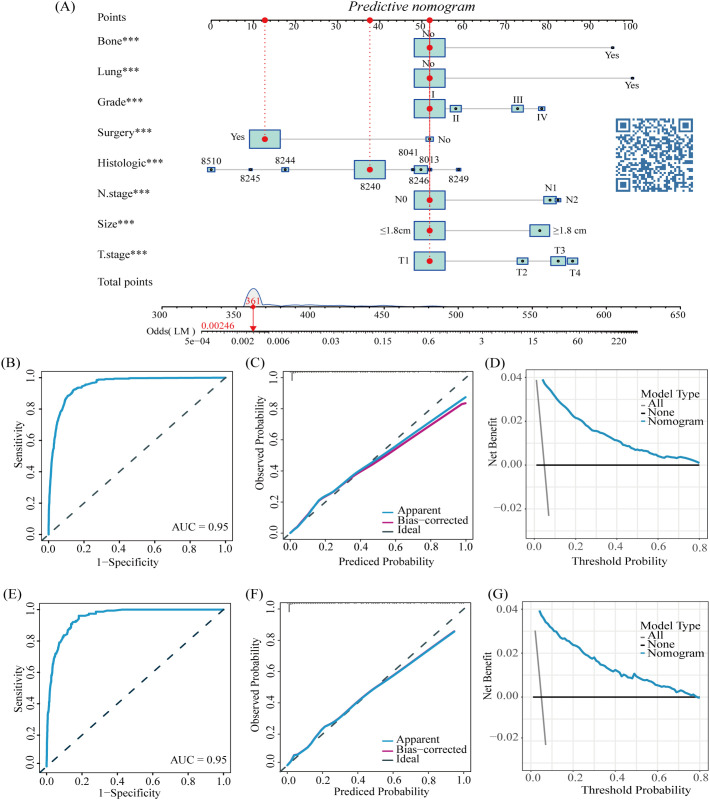
The nomogram for LM in patients with CRNENs **(A)**, and the ROC curves for the predictive nomogram in the training **(B)** and validation group **(E)** Calibration curves in the training **(C)** and validation group **(F)** DCA curves in the training **(D)** and validation group **(G)**. The "*" indicates the significance level of the independent variable. The number of "*" varies when the significance levels are different.

In the published article, there were some language errors. Due to these errors, several mistakes occurred in the original text.

1. A correction has been made to the **Abstract**, *Results:*, Lines 2 to 5.

This sentence previously stated:

“The result of multivariate logistic regression analyses indicated that histologic type, tumor grade, T stage, N stage, lung metastasis, bone metastasis, and tumor size were independent predictive factors for LM in patients with CRNENs (p < 0.05).”

The corrected sentence appears below:

“The result of multivariate logistic regression analyses indicated that histologic type, tumor grade, T stage, N stage, lung metastasis, bone metastasis, surgery, and tumor size were independent predictive factors for LM in patients with CRNENs (p < 0.05).”

2. A correction has been made to **Result**, *3.2 Risk factors and predictive nomogram for LM in CRNETs*, 3.2.1 Risk factors analysis of LM in CRNETs.

This sentence previously stated:

“The results of univariate logistic demonstrate that age, gender, race, histologic type, Grade, Tstage, N stage, lung metastasis, bone metastasis, and tumor size are factors related to the occurrence of LM in patients with CRNENs (Table 3; p < 0.05). Multivariable logistic analysis results revealed that histologic type, Grade, T stage, N stage, lung metastasis, bone metastasis, and tumor size are the independent influencing factors for LM in patients with CRNENs (Table 3). In contrast, the histological type (8240;8244;8510) may be the protective indicator (Table 3; OR < 1;p < 0.05).”

The corrected sentence appears below:

“The results of univariate logistic demonstrate that age, gender, race, histologic type, Grade, T stage, N stage, lung metastasis, bone metastasis, surgery, and tumor size are factors related to the occurrence of LM in patients with CRNENs (Table 3; p < 0.05). Multivariable logistic analysis results revealed that histologic type, Grade, T stage, N stage, lung metastasis, bone metastasis, surgery, and tumor size are the independent influencing factors for LM in patients with CRNENs (Table 3).

In contrast, the histological type (8240;8244;8510) and surgery may be the protective indicator (Table 3; OR < 1;p < 0.05).”

3. A correction has been made to **Result**, *3.2 Risk factors and predictive nomogram for LM in CRNETs*.

This sub-headings previously stated:

“3.2 Risk factors and predictive nomogram for LM in CRNETs

3.2.1 Risk factors analysis of LM in CRNETs

3.2.2 The predictive nomogram for LM in CRNETs”

The corrected sub-headings appears below:

“3.2 Risk factors and predictive nomogram for LM in CRNENs

3.2.1 Risk factors analysis of LM in CRNENs

3.2.2 The predictive nomogram for LM in CRNENs”

4.A correction has been made to **Result**, *3.3 Prognostic factors and nomogram for LM-CRNENs*, 3.3.1 Prognostic factors analysis of LM-CRNENs.

This sentence previously stated:

“Primary tumors originating in the rectum, histologic types 8240 and 8510, surgery, and chemotherapy were considered protective factors in prognosis (Table 4; HR < 1, p > 0.05). ”

The corrected sentence appears below:

“Primary tumors originating in the rectum, histologic types 8240 and 8510, surgery, and chemotherapy were considered protective factors in prognosis (Table 4; HR < 1, p<0.05). ”

The authors apologize for these errors and state that this does not change the scientific conclusions of the article in any way. The original article has been updated.

